# Journey of a stone—thirty years on

**DOI:** 10.1093/jscr/rjad069

**Published:** 2023-02-23

**Authors:** Xinyi Nan, Ramesh Damodaran Prabha, Muddassir Rashid, Bhavik Patel

**Affiliations:** Department of General Surgery, Surgical & Critical Care Division, Gold Coast Health & Hospital Services, Gold Coast QLD, Australia; Hepatobiliary Surgery, Department of General Surgery, Surgical & Critical Care Division, Gold Coast Health & Hospital Services, Gold Coast QLD, Australia; Diagnostic and Interventional Radiology, Gold Coast Health & Hospital Services, Gold Coast QLD, Australia; Acute Care and Trauma Surgery, Gold Coast Health & Hospital Services, Gold Coast QLD, Australia

## Abstract

Laparoscopic cholecystectomy is the gold standard surgical treatment of gallstone disease and a commonly performed procedure in general surgery. Retained gallstones from intraoperative spillage remain largely asymptomatic and complications are rare. Peak incidence of presentation occurs within a year; however, it is important to recognize retained gallstones as a differential for acute presentations even many years postoperatively. We present the case of a 74-year-old female who presented with a retained-gallstone-associated abdominal wall abscess 30 years after spillage during the original surgery, which was successfully treated with a stepwise extraperitoneal approach with local drainage.

## INTRODUCTION

Laparoscopic cholecystectomy (LC) is considered the gold standard surgical treatment of gallstone disease and is one of the commonest performed abdominal surgical procedures [1]. Intraoperative spillage of gallstones can occur with literature citing an incidence of 6–40%, however with low morbidity at 0.08–0.3% [2, 3]. Nevertheless, gallstone-related abscess is a well-recognized complication which can present a diagnostic dilemma given non-specific clinical symptoms and variable time to presentation. We present the case of a 74-year-old female who presented to the emergency department with right flank swelling resulting from a single retained stone that migrated into the abdominal wall 30 years after LC.

## CASE PRESENTATION

A 74-year-old female presented to the emergency department with a 5-day history of intermittent right-sided abdominal pain postprandially and swelling in the right flank. She had no changes in bowel habits or urinary symptoms. Physical exam revealed tenderness to the right flank without guarding, and LC scars from over 30 years ago were noted. Laboratory tests revealed an infective picture with leukocytosis of 13, neutrophilia 8.89 and C-reactive protein of 141. Her past medical history is significant for type-2 diabetes, hypertension, ischaemic heart disease on dual antiplatelet therapy (DAPT), heart failure, dyslipidemia and obesity. Her surgical history includes LC in the 1990s, with the patient noting she was aware that one stone was left behind at the time. She is a Jehovah’s Witness and will not accept any form of blood products.

**Figure 1:**
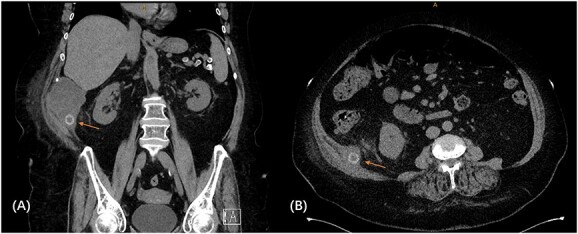
CT scan demonstrating retained gallstone with associated abscess ~62 × 86 × 98 mm in the right flank involving the right posterior transverse abdominis/internal oblique muscle in the coronal **(A)** and axial **(B)** views.

**Figure 2:**
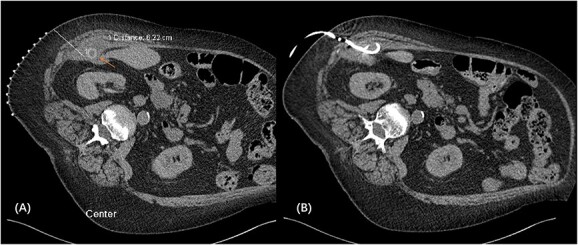
CT-guided pigtail re-insertion pre-operatively to assist localization of gallstone **(A)**; drain placed in the right subhepatic collection located within the transverse abdominis muscle **(B)**.

**Figure 3:**
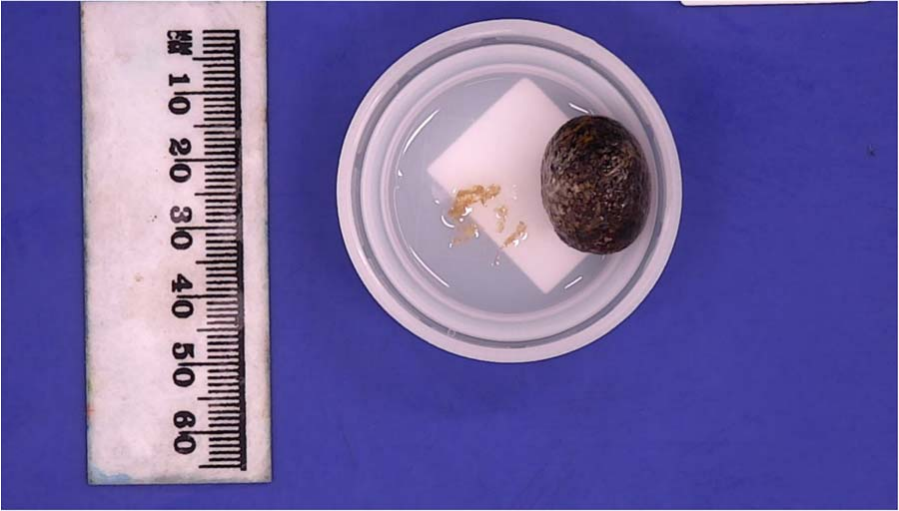
Retrieved gallstone measuring 18 × 15 × 14 mm, weighing 3 g and multiple loose fragments of tan tissue 5× 4 × 1 mm in aggregate.

We proceeded with a multiphase computed tomography (CT) scan which was suggestive of a mixed density lesion measuring 62 × 86 × 98 mm arising from the right posterior transverse abdominis/internal oblique muscle, with a well-defined rounded calcific focus at its inferior aspect likely representing a dropped gallstone (GS) associated with abscess formation (Fig. 1). She was commenced on intravenous antibiotics and, in review of her medical comorbidities and DAPT on board, underwent image-guided percutaneous drainage of the gallstone associated abscess collection in the first instance. Following a course of intravenous and oral antibiotics, she was followed up in the outpatient clinic where the drain was removed after 9 days as the output was serous and minimal. Clearance from anaesthetics and cardiology were obtained during pre-operative evaluation of fitness towards surgery, including an ECHO which demonstrated severe left atrial enlargement and moderate mitral stenosis but normal systolic function. DAPT was withhold since initial admission. She underwent an elective procedure 4 weeks later from the initial presentation with pre-operative CT-guided drain localization of the gallstone (Fig. 2) followed by an extra-peritoneal approach of exploration of the right flank. A single 18 × 15 × 14 mm intramuscular gallstone was removed from a deep intramuscular abscess cavity at 6 cm, with fibrous and inflamed tissue surrounding the stone (Fig. 3). Histopathology demonstrated a gallstone with acute inflammation and acellular debris. The postoperative course was uncomplicated with patient discharged on Day 3 post operation and remained well at outpatient follow-up with DAPT restarted.

## DISCUSSION

LC is a commonly performed procedure in general surgery. Retained GS may remain largely asymptomatic and complications are rare, occurring in a reported 0.08–8.5%, but is well recognized to account for a spectrum of presentation both in the early and late postoperative period [3].

Acting as a nidus for infection, abscess formation from retained GS is a commonly reported complication with literature citing 60% [4]. Most frequently these occur intra-abdominally in peri-hepatic locations but can be widely distributed in the abdomen and pelvis [5]. Abdominal wall abscesses are less frequent and can often occur at previous port insertion sites. Other reported complications include formation of fistula tracts (such as peritoneal-cutaneous, boncho-peritoneal and colonic-cutaneous), bowel obstructions and incarceration in hernia sacs [6, 7]. Presentation time is variable and can occur from a few months to many years post LC, with peak incidence cited to be within a year [4].

Presenting symptoms can be non-specific with fevers and abdominal pain, which often impose a diagnostic challenge when coupled with the variable time to presentation. CT is a commonly used for initial investigation, with high calcium-containing stones identifiable like in this case [5]. Retained GS have the potential to mimic cancer, with reported cases of mistaking them for peritoneal metastatic deposits, lymph nodes or soft tissue sarcomas, leading to oncological workup [5, 8]. Similarly in this case, the gallstone abscess can masquerade as a soft tissue lesion arising from the transverse abdominis with a calcific focus. This highlights the importance of presenting clinically relevant history with imaging requests, and the familiarity of having retained GS as a differential. Recognizing the stone within the abscess is essential for timely definitive treatment.

Source control must be achieved with stone removal to prevent recurrent abscess or fistula formation. In view of the medically comorbid patient with DAPT on board, we utilized a stepwise, multidisciplinary approach working closely with interventional radiology (IR). Initial sepsis was controlled with antibiotics and IR percutaneous drainage, allowing time for further workup towards delayed definitive surgery with preoperative IR localization (Fig. 2) to precisely track the target and minimize the size of the incision. Staying extraperitoneal with local drainage and evacuation is demonstrated to be safe and effective.

LC was first introduced into Queensland in 1990, and this case was one of the earlier performed LCs in the state [9]. The unfortunate spillage of calculi might be secondary to surgeons still on the learning curve related to technical skills needed to perform laparoscopic surgery on inflamed gallbladders. Understandably at the time, there was also limited knowledge of the potential of delayed complications from retained GS. Nonetheless, the importance of avoiding gallbladder perforation and retrieving as many spilled stones as possible has now been well established. This is one of the rare cases to the authors’ knowledge of a 30-year delay for complications secondary to a retained GS which was spilled during the original surgery.

## CONFLICT OF INTEREST STATEMENT

There are no conflicts of interest to declare.

## FUNDING

None.

## DATA AVAILABILITY

Data sharing is not applicable to this article as no new data were created or analyzed in this study.
